# Pathogenic Immunoglobulin A-Producing Cells in Immunoglobulin A Nephropathy

**DOI:** 10.3390/jcm13175255

**Published:** 2024-09-05

**Authors:** Yuko Makita, Heather N. Reich

**Affiliations:** 1 Division of Nephrology, University Health Network, Department of Medicine, Temerty Faculty of Medicine, University of Toronto, Toronto, ON M5S 1A1, Canada; yuko.makita@uhn.ca; 2 Department of Nephrology, Juntendo University Faculty of Medicine, Tokyo 113-8421, Japan; 3 Toronto General Hospital Research Institute, Toronto, ON M5G 2C4, Canada

**Keywords:** IgA nephropathy, mucosal immunity, APRIL/BAFF, genetic factors

## Abstract

Immunoglobulin A nephropathy (IgAN) is the most prevalent primary glomerular disease worldwide and it remains a leading cause of kidney failure. Clinical manifestations of IgA are exacerbated by infections, and emerging data suggest that aberrant mucosal immune responses are important contributors to the immunopathogenesis of this disease. However, the exact stimuli, location and mechanism of nephritis-inducing IgA production remains unclear. In this focused review we explore recent developments in our understanding of the contribution of the mucosal immune system and mucosal-derived IgA-producing cells to the development of IgAN.

## 1. Introduction

Immunoglobulin A nephropathy (IgAN) is the most common primary glomerular disease in many countries and progresses to end-stage kidney disease (ESKD) in approximately 40% of patients within 15 to 20 years after onset [1]. It has been more than 50 years since the first report of IgAN, yet many aspects of the pathogenesis of the disease remain unknown, and disease-specific curative treatments do not exist.

The prevalence of IgAN exhibits regional variation [2]. For example, IgAN accounts for 54% of biopsy-proven diagnoses in cohorts from China [3], and 7% of adult biopsies from American cohorts [4]. These regional differences may be, in part, attributed to variations in criteria for routine population urine screening and renal biopsy indications. In Pacific Asian countries, routine practice often involves performing renal biopsies in patients presenting with persistent microscopic haematuria, with or without proteinuria, whereas the threshold for diagnostic biopsy is different in other global healthcare systems. Differences in screening and biopsy practice, however, do not fully account for variation in disease incidence and prevalence. Genetic factors are strongly associated with susceptibility to IgAN, and the interaction of environmental influences and genetic susceptibility is an important area of investigation [5]. 

The diagnosis of IgAN requires a kidney biopsy finding of mesangial IgA deposits by immunostaining. Characterization of circulating and tissue IgA-containing immune deposits has further elucidated some of the potential steps required for the development of this glomerular disease. The prevailing framework to describe the immunopathogenesis of IgAN includes four key putative “hits” [6]. First, patients with IgAN exhibit increased production of galactose-deficient IgA1 (Gd-IgA1), which is detectable in the circulation in polymeric form [7,8]. Second, IgG auto-antibodies are formed targeting the under-galactosylated hinge region of the Gd-IgA1 [9]. Third, these Gd-IgA1 and IgG auto-antibodies form circulating immune complexes, often containing components of the alternate pathway of complement activation, including C3. The fourth hit involves the deposition of these “nephritogenic” immune complexes in the glomerular mesangium, triggering local cellular proliferation and inflammation, ultimately resulting in glomerular sclerosis and interstitial fibrosis. 

The site and trigger for production of Gd-IgA1 centrally implicated in IgAN remain unknown. Two dominant but not necessarily mutually exclusive hypotheses have been investigated. One line of evidence supports the idea that the cells producing pathogenic disease-causing IgA are located within the mucosal associated lymphoid tissue. Alternatively, a body of work suggests that pathogenic IgA is produced by mucosal-derived B cells located in the bone marrow. In this article we will review the evidence supporting these hypotheses and explore new research from other diseases suggesting that immune modulatory IgA-producing cells may be located in previously unappreciated tissues and anatomic compartments. In this review, we focus specifically upon the production of pathogenic IgA and novel therapeutic approaches to interrupt this process. 

## 2. Production and Characteristics of IgA

Approximately 3 g of IgA is produced daily in humans, and this accounts for three quarters of total immunoglobulin production [10]. Most IgA production occurs in the mucosa-associated lymphoid tissue (MALT), which lines the gastrointestinal tract (GALT), nasopharynx (NALT), bronchi (BALT) and other mucosal surfaces. The IgA produced by the MALT is targeted towards secretion across the epithelial barrier at mucosal surfaces, into the mucous coating, where it serves as a primary defence against exposure of the host bloodstream to foreign antigens and microbes. In addition to the trapping of bacteria within the gut for subsequent elimination, coating of microbes and dietary antigens with IgA at mucosal surfaces also tempers the host immune response to mucosal contents, and is therefore important to the maintenance of host–microbiome mutualism [11,12].

Humans produce two subclasses of IgA—IgA1 and IgA2. These subclasses are structurally different, primarily due to variation in the hinge region, with differing numbers of glycosylation sites. Emerging data suggest that these subclasses also have different effector functions modulating immune cell activation, with distinct binding and signaling properties [13]. In the serum, 90% of circulating IgA is of the IgA1 subclass, and 10% is IgA2; these ratios differ across mucosal tissues and secretions [10]. While IgA in the circulation is primarily monomeric, the IgA found in mucosal secretions often exists as dimers or polymers formed when a heavy chain from one monomer is linked to a heavy chain on a separate monomer by a small glycosylated peptide known as a “Joining” or J chain [14,15]. Secretory IgA (S-IgA), most often dimeric in structure, is the primary Ig subtype found in milk, saliva, and secretions lining mucosal surfaces, such as those found in the respiratory and gastrointestinal tracts. In order to transport locally produced dimeric IgA to mucosal surfaces, dimeric J chain-containing IgA is first bound to the polymeric immunoglobulin receptor (pIgR) at the basolateral side of the epithelium, which lines the mucosal surface. The glycoprotein-binding portion of the receptor is subsequently cleaved, releasing the secretory component (SC), which supports the stability of the IgA dimers, enabling transcytosis to the mucosal surface [16]. Secretory IgA is therefore comprised of the IgA dimers, J chain and SC. 

Production of IgA in healthy subjects is organized into functional anatomic compartments. Classic sites of induction of mucosal immune responses in the GALT—the largest component of the mucosal-associated lymphoid tissue—include the Peyer’s patches and the mesenteric lymph nodes [17]. Antigens derived from food, and commensal and pathogenic microbes located at mucosal surfaces are shuttled to subepithelial myeloid cells by microfold M cells located interspersed within the enterocyte layer [18,19]. Dendritic cells that have sampled antigens then participate in T cell-dependent and independent IgA class switch [20]. In T cell-dependent IgA class switch, CD40 on B cells is engaged by CD40-ligand expressed by T cells activated by dendritic cells within germinal centres, ultimately leading to the expression of NFkB that drives transcription of the enzyme required for class-switch recombination, activation-induced cytidine deaminase [21]. In the presence of locally produced cytokines such as BAFF and APRIL, NFkB-mediated class-switch recombination of B cells can occur independently of T cells [22].

The IgA-expressing B cells activated in the GALT further differentiate to become short-lived IgA-secreting plasmablasts capable of proliferation. These plasmablasts leave the mucosal priming site and circulate through the lymphatics and bloodstream, ultimately migrating back to the intestinal lamina propria—considered a “classical” effector site for IgA^+^ PCs [23]. The niches of IgA^+^ PC may persist within the lamina propria for years, contributing to critical local defence against exposure of the host to invasion by commensal microbes. It is increasingly appreciated that these gut-derived IgA^+^ plasmablasts and PCs may also migrate out of the lamina propria to establish effector niches at other anatomic sites such as the liver and the central nervous system, where they may participate in modulating local inflammatory responses [24].

## 3. Qualities of Pathogenic IgA

The IgA1 subclass possesses a site for *O*-linked glycosylation at its hinge region. Studies using IgA1-producing cell lines derived from the peripheral blood of patients with IgAN have shown an increased proportion of galactose-deficient *O*-glycans in the hinge region [7,8]. The abnormal patterns of glycosylation of galactose-deficient IgA1 (Gd-IgA1) confer self-aggregation and antigenicity to IgA1, facilitating the formation of polymeric IgA or IgA-IgG immune complexes. Furthermore, IgA1 with abnormal glycosylation has been shown to be more readily deposited in cultured mesangial cells [25]. 

Patients with IgAN exhibit elevated serum levels of Gd-IgA1 in proportion to total IgA, and these elevated levels have been associated with decreased renal function [26,27]. The association between elevated Gd-IgA1 and increased risk of renal disease progression [26] suggests that Gd-IgA1 may be a potential prognostic biomarker for IgAN. However, it is important to note that while Gd-IgA1 is elevated in the serum of patients with IgAN, it has also been observed to be elevated in blood relatives of patients without nephropathy [28]. This finding suggests that Gd-IgA1 alone does not fully explain the pathogenesis of IgAN; other pathologic factors are required for the development and progression of the disease. 

In healthy subjects, circulating IgA1 is typically monomeric. Patients with IgAN have increased serum levels of immune complexes containing polymeric IgA1, and the polymeric IgA immune complexes may contain the secretory component [29]. The mesangial and circulating immune complexes in IgAN contain polymeric galactose-deficient IgA [8] and J chain [30]. These observations have supported hypotheses that pathogenic IgA in patients with IgAN is mucosal in origin, or it is produced by IgA-secreting cells that are derived from the mucosal-associated lymphoid tissue but reside within the bone marrow. 

While beyond the scope of this review, the properties of pathogenic IgA (aberrant glycosylation, polymerization) have been proposed to promote the activation of complement via the alternate pathway which perpetuates glomerular and possibly tubulo-interstitial injury in IgAN. While the relative importance of systemic versus kidney-specific complement activation is not known, galactose-deficient IgA1- stimulation of mesangial cells in vitro induces C3 secretion and mesangial proliferation [31]. Disruption of complement activation that may be a consequence of pathogenic Gd-IgA1 production is therefore a strategy for treatment of IgAN, which is an active area of investigation [32]. 

## 4. Where Is Pathogenic IgA Produced?

Since the discovery of IgAN, the location and characteristics of the antibody-secreting cells producing pathogenic IgA has been debated. While the hypotheses are not necessarily mutually exclusive, debate has focused primarily around the relative contribution of aberrant humoral vs. mucosal IgA responses. The responses to both mucosal and systemic immunization appear to be altered in patients with IgAN. Systemic immunization of patients with IgAN with tetanus toxoid is characterized by a an enhanced and sustained increase in circulating polymeric IgA [33], and patients with IgAN also exhibit enhanced humoral responses to measles vaccination [34]. Oral (mucosal) vaccination against polio virus in previously vaccinated patients with IgAN is also characterized by an augmented IgA-biased response [35]. These early observations led to studies aiming to better localize putative cells producing pathogenic IgA, with a focus on distinguishing a humoral vs. mucosal source. 

## 5. Pathogenic IgA Production in Bone Marrow

Early lines of evidence suggested a humoral source for pathogenic IgA. The bone marrow of patients with IgAN contains a higher proportion of IgA1-producing plasma cells, and cultured bone marrow cells from these patients also exhibit enhanced IgA1 production [36]. It was subsequently observed that duodenal biopsies of patients with IgAN demonstrate reduced levels of J chain mRNA-positive IgA plasma cells, supporting the fact that the intestinal mucosa is not the primary source of pathogenic immune complexes containing IgA and J chain observed in IgAN [37]. Subsequently, the same group documented a higher proportion of B cells producing IgA and expressing J chain mRNA in the bone marrow of patients with IgAN [38]. To reconcile these findings, it was proposed that IgAN is characterized by a derangement in the mucosa–bone marrow axis. Mis-homing of B cells initially primed in the mucosal-associated lymphoid tissue results in their deposition in the bone marrow and subsequent differentiation to IgA^+^ PCs within the marrow, where they produce the bulk of pathogenic polymeric IgA1 [39]. 

## 6. Pathogenic IgA Production in the Gut-Associated Lymphoid Tissue in IgAN

Gross haematuria concurrent with upper-airway and gastrointestinal infections is a hallmark of IgAN. As discussed previously, the physical characteristics of circulating and mesangial immune complexes in IgAN suggest that they are mucosally derived. These observations prompted investigations to determine if mucosal-associated lymphoid tissue is the primary source of pathogenic IgA. As the GALT is the largest component of the mucosal immune system, it has naturally been a focus of interest. 

Experimental models of IgAN support an intestinal mucosal source for pathogenic IgA production, with gluten derivatives as a possible trigger. In BALB/c mice, wherein Th2 cells are easily triggered by immunization, oral administration of a series of exogenous dietary antigens induces mesangial IgA deposition [40]. Similarly BALB/c mice placed on a gluten-containing or gliadin-enriched diet display increased anti-gliadin-specific IgA in the serum and glomerular deposit eluates [41]. Mice expressing human IgA1 and CD89 with induced gluten sensitivity develop mesangial IgA1 immune complexes, haematuria, enhanced intestinal IgA1 secretion, and increased serum IgA1 anti-gliadin antibodies, which correlates with proteinuria [42]. In contrast, the mice fed a gluten-free diet for three generations showed a significant reduction in mesangial IgA1 deposition and haematuria [42]. 

As with all human diseases, experimental mouse models are not always ideally suited to fully study pathogenesis in humans. In the case of IgAN, there are important limitations to these models. An obvious limitation is that only primates express the IgA1 subclass. The hinge region of human IgA1 contains O-linked glycans; however, murine IgA has N- but not O-glycans [43]. Therefore the specific impact of increased Gd-IgA1 can only be evaluated if the mouse is genetically engineered to express this human IgA subtype. In humans, IgAN is a clinically and pathologically heterogeneous disease, and it is difficult to capture heterogeneity in experimental models.

Indeed, the experimental observations have not consistently translated to human studies. Dietary antigens are typically not present in circulating IgA-containing immune complexes in IgAN. Furthermore, increased titres of antibodies targeting celiac disease-associated antigens gliadin and transglutaminase-2 are not typically observed in patients with IgAN [44]. In a small uncontrolled study, 29 subjects with IAN adhered to a minimum of 6 months of a gluten-free diet [45]. There was a significant reduction in circulation in IgA-containing immune complexes and levels of IgA targeting gluten antigens. Proteinuria did fall with gluten restriction, although subjects still demonstrated a loss of kidney function, suggesting a lack of clinical impact of a relatively brief period of dietary modification. 

Clinical data do support a link between intestinal inflammatory conditions and development of IgAN. Experimental studies in mouse models have shown that overexpression of LIGHT, a ligand for the Lymphotoxinβ receptor (LTβR), which is involved in mucosal lymphoid tissue formation and IgA production, induces severe intestinal inflammation and an IgAN-like phenotype [46]. These findings suggest that chronic intestinal inflammation, as seen in inflammatory bowel disease, may contribute to the transfer of IgA into the bloodstream and the subsequent development of IgAN. However intestinal inflammation and increased intestinal permeability are not consistently observed in patients with primary IgAN, although findings are confounded by variation in the techniques used to measure these parameters [47]. 

A recent study demonstrating the effectiveness of therapy targeting mucosal IgA production in reducing the clinical disease severity of IgAN underscores the importance of the gastrointestinal-associated lymphoid tissue in IgAN [48]. The proprietary encapsulation of nefecon allows pH- and time-dependent release of budesonide at specific target sites in the gastrointestinal tract [49], particularly in the distal ileum and proximal cecum. This targeted release mechanism ensures that the active compound is delivered to the target site—Peyer’s patches—maximizing local effect while minimizing systemic exposure. Any absorbed drug largely undergoes first-pass metabolism primarily through the cytochrome P450 3A4 pathway, resulting in approximately 90% metabolism of the active drug and reduced systemic exposure [50]. This local action minimizes the potential side effects associated with systemic corticosteroid use. 

The phase 3 randomized placebo-controlled clinical trial demonstrated a marked reduction in proteinuria and attenuation of loss of GFR in patients who received nefecon [48]. Subsequent translational studies indicate that nefecon produces a significant reduction in serum levels of IgA1, Gd-IgA1 and IgA-/IgG containing immune complexes [51]. The mechanisms by which nefecon causes a reduction in these antibodies requires further study; however, a reduction in circulating levels of B-cell activating factor (BAFF) and B-cell maturation antigen (BCMA) support potential effects on pathways implicated in activation and class switch of IgA-producing cells [51]. 

Systemic corticosteroids remain a mainstay of therapy for IgAN, given the low cost of this medication and limited access to nefecon in many countries. Treatment with systemic corticosteroids reduces proteinuria and the risk of kidney failure in IgAN, as demonstrated in a large randomized trial [52,53]. While the IgAN-specific data regarding the impact of systemic corticosteroids on markers of disease pathogenesis such as Gd-IgA1 are limited, it is well established that glucocorticoids impact B cell development and survival [54], in addition to the known general anti-inflammatory properties. Activity at the level of the Peyer’s patches is plausible. The main limitation to systemic corticosteroids is the undesirable side-effect profile, which limits tolerability and can result in serious toxicity at high doses or with prolonged exposure. 

The direct action localized to the Peyer’s patches distinguishes nefecon from systemic corticosteroids. While it is difficult to precisely model the systemic absorption, analyses based on the suppression of endogenous cortisol suggest that 16 mg of Nefecon would result in the approximate level of suppression of 8 mg of prednisolone [55]. This supports the concept that it would have a favourable toxicity profile compared to systemic glucocorticoids. Following treatment with both systemic glucocorticoids and nefecon, proteinuria does tend to return, even in patients who ultimately derive benefit from the intervention with respect to GFR loss. The important impact of nefecon on IgAN clinical parameters highlights the role of the GALT in IgAN pathogenesis, yet there remains an unmet need for an intervention that provides a longer remission or is sufficiently safe to be given for an extended period or in repeated cycles. The impact of repeated dosing of nefecon should be evident once the results are available from the open-label extension study that followed the core phase 3 study of nefecon. 

## 7. Involvement of Nasal-Associated Lymphoid Tissue (NALT)

The association of IgAN with gross haematuria after upper respiratory tract infection suggests that the involvement of an abnormal mucosal immune response triggered by foreign antigens exacerbate IgAN. In Japan, the successful routine use of tonsillectomy combined with steroid pulse therapy to treat IgAN reflects the support and the important contribution of the NALT tonsillar tissue on the development of this disease [56,57]. The Japanese Nationwide Retrospective study in IgAN, an observational study examining the relationship between tonsillectomy and renal outcome in patients with IgAN, reported that patients who underwent tonsillectomy within 1 year of renal biopsy had a decreased risk of renal function decline at 10 years [58]. In addition, decreased serum levels of Gd-IgA1 and improved urinary findings have been observed after tonsillectomy [59], further supporting the involvement of mucosal exposure to NALT and exogenous antigens in the development of IgAN. 

Several studies have highlighted differences in the composition of the bacterial flora of the tonsils, saliva, and subgingival marginal region between IgAN patients and controls. Although investigations of mucosal immunity and microbiota in IgAN have not identified common antigens, associations with several tonsillar bacteria, including *Haemophilus parainfluenzae*, *Staphylococcus aureus*, *Neisseria* sp., and *Prevotella* sp., have been observed in patients with IgAN [60,61,62]. 

The data supporting a role for tonsillectomy in IgAN have largely been derived from Japanese cohorts. The benefit of tonsillectomy has not been consistently observed in other populations. For example, a retrospective study of 41 Caucasian European patients who underwent tonsillectomy did not suggest improved renal outcome compared to propensity-matched subjects [63]. It is unclear whether there may be a differential benefit of tonsillectomy based upon ancestry or geography. Studies performed outside of Japan are retrospective and small in size, making definitive conclusions in non-Japanese cohorts challenging. 

Accumulating evidence supports the idea that abnormal mucosal immune responses in GALT and NALT contribute to the development and progression of IgAN. The efficacy of locally-active budesonide and tonsillectomy and the observed differences in bacterial flora in patients with IgAN further emphasize the contribution of mucosal exposure and exogenous antigens in the disease process (see previous section).

## 8. The Role of APRIL and BAFF in IgAN

Genome-wide association studies conducted in a cohort of 4137 Chinese patients revealed that the *TNFSF13* locus is a disease-susceptibility gene for IgAN [64]. This gene encodes A proliferation-inducing ligand (APRIL), a member of the tumour necrosis factor superfamily with high homology to B-cell activating factor (BAFF). 

The development of the adaptive immune response requires the maturation, selection, and expansion of B cells to generate protective antibodies targeting foreign antigens. The cytokines APRIL and BAFF are produced by monocytes, macrophages and dendritic cells, and contribute to T-cell independent B cell differentiation, proliferation, antibody production, and class switch [65]. While these cytokines are both important for B cell maturation and survival, they differ in terms of receptor specificity and activity. The BAFF cytokine binds to the BAFF receptor (BAFF-R), the B cell maturation antigen (BCMA) receptor, and the transmembrane activator and calcium modulator and cyclophilin ligand interactor (TACI) receptor. Through these interactions, BAFF impacts largely earlier B cell development, and maturation [66]. APRIL binds strongly to BCMA and, with lower affinity, to the TACI receptor, generally impacting later stages of B-cell and plasma-cell maturation and survival, including immunoglobulin class switch, as discussed above.

Experimental evidence supports a central role for BAFF and APRIL in tonsillar responses to microbial antigens [67,68]. In a mouse model of IgAN, the administration of a neutralizing-antibody targeting APRIL resulted in the suppression of glomerular IgA deposition and improved nephropathy, underscoring the significance of APRIL in disease progression [69,70].

Recent clinical studies support a role for APRIL and BAFF in the pathogenesis of IgAN. Multiple studies demonstrate increased serum levels of BAFF and APRIL in patients with IgAN compared to healthy controls, and levels of these cytokines positively correlate with disease severity (Review [71]). Recently, this association has entered the clinical realm [71]. Emerging data indicate that interruption of APRIL signalling—through targeted blockade of circulating APRIL or the TACI receptor—has direct impact on biologic and clinical markers of disease activity. Sibeprenlimab is a humanized IgG2 monoclonal antibody that binds and neutralizes APRIL. Interruption of APRIL signalling with this approach results in reduction in levels of pathogenic Gd-IgA1, as well as total IgA, IgM and IgG [72]. In a large phase II study, treatment with sibreprenlimab resulted in a reduction in proteinuria, and stabilization of renal function at one year [72]. The phase 3 study is underway (NCT05248646), and will provide information regarding the impact of 2 years of treatment on proteinuria and GFR decline. The use of zigakibart, another inhibitor of soluble APRIL is also being studied in a phase 3 trial (NCT05852938), with encouraging interim results regarding proteinuria reduction from phase 1/2 trial data [73].

An alternate approach to the inhibition of APRIL by the production of pathogenic IgA is blockade of the TACI receptor using TACI-fusion protein antagonists, impacting both BAFF and APRIL signalling. Telitacicept [74] is already approved for the treatment of lupus in China, and is in phase 3 clinical development in IgAN (NCT05799287). Similarly blockade of the TACI receptor with Atacicept has been applied across several auto-immune diseases. Phase 2 data support a reduction in Gd-IgA1 in patients with IgAN and significant reduction in proteinuria, with phase 3 development underway [75] (NCT04716231). Finally the fusion protein TACI antagonist povetacicept is in development for auto-immune diseases including IgAN (NCT05732402). The relative benefits and risks of directly inhibiting APRIL versus the combination of effects of BAFF and APRIL signalling through the TACI receptor will likely only be fully appreciated once phase 3 clinical trials are complete. 

## 9. Clues from Genetic Studies in IgAN

The variation in the geographic and ethnic distribution of IgAN suggests that genetic factors contribute to disease pathogenesis. Genome-wide association studies have consistently identified susceptibility loci within genes encoding the major histocompatibility complex (MHC), as well as within interferon-related genes and genes associated with regulation of innate immune function [76,77,78]. 

Those detected as risk genes associated with IgAN in this cohort were also found to overlap with disease-related genes for autoimmune and inflammatory diseases such as rheumatoid arthritis, scleroderma, IgA deficiency, SLE, ulcerative colitis, and inflammatory bowel disease [76]. In particular, certain genes, including ITGAM and TNSF13, are associated with mucosa-associated lymphoid tissues involved in IgA production, while Fc receptor (FcR), CARD9, VAV3, PSMB8, and PSMB9 are associated with NF-κB activation and the mucosal barrier formed by intestinal epithelial cells [76]. These findings strongly suggest that mucosal immunity is involved in the genetic aetiology of IgAN. Disease-associated gene overlap with inflammatory bowel disease further supports a role for mucosal immunity in this condition.

## 10. The Role of IgA-Producing Cells at Extramucosal Sites

The complex relationship between pathogenic IgA production and abnormal mucosal immunity in two pivotal sites, NALT and GALT, is a focus of research on the immunopathogenesis of IgAN. It has recently been appreciated that gut-derived signals can regulate peripheral extramucosal immune responses with important disease-modifying effects through IgA production [79]. This is one manner in which mucosal microbiota and dietary antigens may affect immune responses at tissues remote from mucosal sites of induction of immune responses. 

The mucosal immune system of both mice and humans is critically involved at the interface of preventing invasion of pathogenic bacteria, fostering immune tolerance and shaping communities of commensal flora at mucosal surfaces. In turn, the mucosal microbiota also shape host immune responses including IgA B cell repertoire. Mucosal or systemic microbiota exposures shape the B cell repertoire [80]. Recent studies suggest that mucosal exposures to commensal flora may impact immune and inflammatory responses at remote sites, including the brain. In a mouse model of experimental multiple sclerosis (MS) it was confirmed that commensal-reactive IgA-producing cells migrate to inflamed sites in the brain and spinal cord in the context of autoimmune encephalomyelitis (EAE). Furthermore, these migrating IgA-producing cells were shown to play a role in *suppressing* inflammation through the production of IL-10 [24]. 

In a subsequent study, IgA-producing cells specific for microbial taxa over-represented in stool samples of patients with MS were identified in the cerebrospinal fluid of patients with active disease [81]. In postmortem brain samples, these microbial-targeted IgA-producing cells were localized to inflammatory lesions in patients with MS and other inflammatory neurologic diseases. These cells were CD138^+^ and CD19^+^ but not CD20^+^, suggesting a plasma cell phenotype, and they were also noted to express IL10, suggesting a protective role, as described in the experimental model [24,81].

The potentially protective role for IgA-producing plasma cells in active MS may explain the surprising results of a clinical trial of a novel approach aiming to reduce neuro-inflammation in MS. A phase II clinical trial investigating the use of atacicept to prevent BAFF and APRIL signalling through the TACI receptor to treat MS was halted prematurely, due to safety concerns. Patients receiving atacicept demonstrated a paradoxical increase in radiologic evidence of disease relapse [82]. At the time of this study, the findings could not be explained, as it was generally expected that modulating TACI signalling would result in a reduction in pathogenic IgG production by B cells. Given the findings that commensal reactive IgA-producing plasma cells localized to the brain in active MS may be protective, the negative impact of therapeutically suppressing these cells may now be explained. 

## 11. IgA-Producing Cells in the Kidney

**Figure 1:**
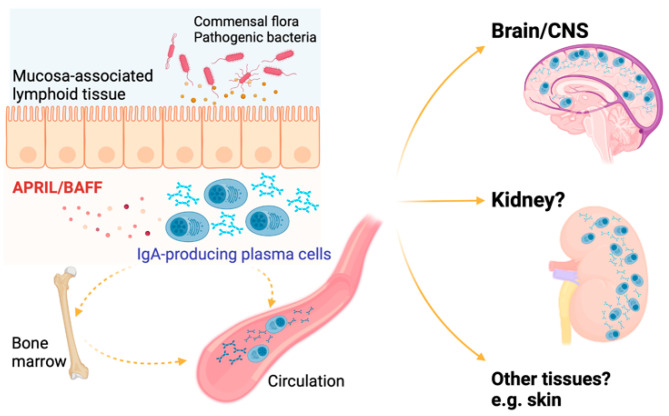
**Mucosa-derived IgA antibody-secreting cells migrate to locations outside of the mucosal compartment.** In the presence of B-cell activating cytokines APRIL and BAFF, mucosal-primed IgA-producing plasma cells may localize to non-mucosal tissues, including typically immune privileged sites such as the central nervous system. Figure created with BioRender.com.

The ability of IgA-producing cells to migrate to inflammatory tissues and accumulate in mucosal tissues is of potential interest in the pathogenesis of IgAN. A recent study used a similar translational approach to look at the role of commensal-reactive IgA in IgAN. In this study, authors noted a higher rate of tonsil colonization by *Neisseria* in IgAN patients compared to healthy controls [60]. The patients in this study had elevated levels of serum IgA, which specifically targets commensal and pathogenic *Neisseria* species [60]. The authors then reverse-translated this observation in an experimental IgAN model, the BAFF-transgenic mouse. Overexpression of BAFF (BAFF-Tg) in this model promotes IgAN-like kidney disease, dependent on the presence of commensal bacterial flora [83]. The authors rendered the BAFF-Tg mice susceptible to infection with *Neisseria*, and demonstrated that mucosally primed anti-*Neisseria*-specific IgA-secreting cells cluster within the kidneys of these mice [60]. These findings suggest that an abnormal cytokine-driven mucosal immune response to oropharyngeal organisms such as *Neisseria* is involved in the immunopathology of IgAN. In the presence of excess BAFF, pathobiont-specific IgA-producing cells may migrate into the kidney to produce IgA, which contributes to development of nephritis (Figure 1). Further delineation of the phenotype of these cells and the mechanisms that facilitate their migration to remote sites might provide new insights into the pathogenesis of human IgAN. 

The reason for the particular affinity of mesangial cells for pathogenic Gd-IgA binding is an area of evolving knowledge. A recent study demonstrated that in the grouped ddY mouse, a spontaneous IgAN model, pathogenic IgA targeting mesangial antigens is found in the serum [84]. Specifically these mice produce IgA against βII-spectrin, a protein typically expressed within the cytoplasm of mesangial cells, but present on the surface of these cells in this experimental model. The authors then determined that 85% of patients with IgAN in their cohort have circulating IgA targeting mesangial lysate protein components, including IgA1 antibodies against βII-spectrin. In the mouse model, IgA^+^ CD138^+^ plasmablast-type cells could be identified by flow cytometry in the kidneys of these mice, and IgA antibodies cloned from these plasmablasts bind to the surface of mesangial cells [84]. This is a second line of evidence supporting the hypothesis that disease-causing IgA-producing cells targeting neoantigens may be localized to the kidneys.

## 12. Factors Fostering Migration of IgA-Producing Cells

Integrin and chemokine signalling are important for the directional migration of IgA-producing cells and may be therapeutic targets to promote or block migration of these cells to mucosal and extra-mucosal sites. Chemokine receptors CCR9 and CCR10, along with integrins, are important for selective homing of IgA-producing cells to the GALT and other mucosal epithelial tissues [85]. Specifically, the chemokine TECK/ CCL25, secreted by gut epithelial cells, binds to CCR9 on IgA-producing cells to facilitate their migration to the intestine [85]. MAdCAM-1 on intestinal endothelial cells also aids in recruiting integrin α4β7-expressing IgA-producing cells homing to the intestine [85].

Similarly, the interaction between MEC/CCL28 and CCR10, and between VCAM1 and integrin α4β1, is vital for the migration of IgA-producing cells to tissues outside of the GALT [86,87]. In mice, MEC/CCL28 upregulation in lactating mammary-gland endothelium attracts CCR10-expressing IgA-producing cells [87], while VCAM-1 on nasal mucosa endothelium engages α4β1-expressing cells [86]. These mechanisms suggest that IgA-producing cells expressing CCR10 and α4β1 are specifically directed to non-intestinal mucosal sites.

In patients with IgAN, Gd-IgA1-producing cells with high levels of CCR9, CCR10, and α4 expression contribute to local antibody production during infections [88]. Based on these observations, one can hypothesize that Gd-IgA1-producing cells expressing these receptors home to the upper respiratory tract, gastrointestinal tract, and secondary lymph nodes or other tissues. Overall, the migration of IgA-producing cells driven by the expression of specific receptors and chemokines plays an important role in IgAN and mucosal immune responses. Further studies are needed to explore the precise mechanisms underlying IgA-producing cell migration to non-mucosal tissues and the role of recruited extramucosal IgA-producing cells in the pathogenesis of IgAN.

The potential importance of plasma cells in the pathogenesis of IgAN, whether located at the level of the gut or at extramucosal sites, supports the exploration of the use of plasma-cell targeted therapies for this disease. Proteosome inhibition with drugs such as bortezomib is associated with significant off-target toxicity with prolonged exposure, but short-term exposure may show clinical effect on proteinuria in IgAN [89]. The development of a novel approach to target CD38-positive plasma cells using felzartamab is being explored in IgAN NCT05065970.

## 13. Conclusions

Clinical observation of synpharyngitic haematuria first suggested that IgAN is a disease of the mucosal immune system. This was later supported by observing that the physical characteristics of pathogenic IgA more closely resemble mucosal-produced antibodies and immune complexes. The characteristics and location of cells producing pathogenic IgA has been debated; however, data are now converging to support the fact that these cells are of mucosal origin, and that mucosal-primed IgA-producing plasma cells may be contributing to disease in extramucosal tissues.

Better understanding of the source of pathogenic Gd-IgA1 production has led to innovation and advances in therapeutics that directly target the GALT, BAFF-APRIL signalling, and IgA-producing plasma cells as a key proponents of pathogenic IgA production.

Further exploration to understand the factors that foster activation and migration of cells that produce pathogenic IgA promises to elucidate additional novel treatment approaches to improve the outcome of patients with this disease. It is confirmed that the clinical improvement noted with new therapeutics relates to fundamental alterations in B cell development or migration is challenging, given limited non-invasive markers beyond general serum and urine inflammatory markers and Gd-IgA1 measurements. Sensitive tissue-based molecular and histologic studies are likely required to establish the impact of these interventions in human subjects. Ultimately, patients with high risk of disease may require treatment using a multi-target approach to optimize outcomes and minimize treatment side effects.
